# Homœopathic Physicians and Regular Medical Societies

**Published:** 1875-01

**Authors:** 


### Homeopathic Physicians and Regular Medical Societies.

Some months since an article appeared in one of the Buffalo daily papers describing two important cases which had come under the care of a Homeopathic practitioner, the writer concluded by asking if the Editor of the Buffalo Medical Journal could tell why this physician was denied admission to the Erie County Medical Society. We did not at the time consider the communication worthy of reply, especially when another practitioner of the same school complained that the author of the letter had been stealing his thunder.

The recent action of the Massachusetts Medical Society in expelling some of its members for practicing homeophathy, has revived the old cry of persecution, and has attracted considerable attention from various quarters.

A correspondent of the *Springfield Republican* of December 20th, shows such a good apprecation of the situation, that we reproduce part of his letter in these columns, and consider it a very fair reply to "Homo," as it emenates from a non-professional source.

BOSTON, Thursday, December 24.

THE HOMEOPATHIC TRIALS

seem narrowed down to a very small point, if one may judge from the defense of Dr. Clapp, one of the accused. He states, what I suppose will be admitted to be true of the best class of doctors of his school, that the honest and intelligent among them do not pretend to be confined to a system or dogma; and that, as far as they do, they claim scarcely more than that in a majority of cases their "system," if it may be so called, is preferable to the old "system." On the other hand, the allopathic or old school, or regular,—no matter what he is called or calls himself,—points to the rule of his society that no person shall be admitted a member who proposes to cure by Spiritualism, Homeophathy or Thompsonianism. As between two physicians, this does not preclude any man from believing that a doctor who calls himself a Spiritualist, Thompsonian or Homeopathist may be more useful than an M. D., according to membership in the Medical Society,—for the former may have experience or insight or science to find out where the malady lies, which is certainly the most important branch of the profession. Ought not, therefore, the scientific homeopathic physician to be honest enough to forego the temptation to take a name which leads men to a superstitious respect for him, based on a worthless dogma? But the case is not a rare one in any relation of life especially of professional life. A young fellow with slim prospects for a subsistence, no experience beyond the treatment of the ordinary belly-ache of New England, finds himself obliged to settle in a growing town and unsettle the health of its inhabitants. Some of the leading people he finds attracted toward the new quackery out of disgust for the old heroic routine. So he says he is of that school, and he persuades the mother that there will be fewer colics and wry faces around the domestic hearth and bed-side if she fills up the "mankle-shelf" with *his* tumblers and spoons, and sends the old physician afloat with his saddle-bags. He succeeds to the practice; if he is a student and a man of brains he gradually, without leaving the taking names, leaves the dogma and the system, and becomes a safe person to have in the house; if an ass, as is pretty often the case, he keeps the grave-yard well populated—the children and women in child-bed being his easiest and most interesting and helpless victims. It is hard to tell whether such rogues had better be let alone or exposed. The infatuation which gives them a living is hard to cure, any way. I do not believe the "persecution" to which they are now subjected is, on the whole, unjust, however unwise it may be.—"*Warrington.*"

